# SOCS3 Genetic Polymorphism Is Associated With Clinical Features and Prognosis of Hepatocellular Carcinoma Patients Receiving Hepatectomy

**DOI:** 10.1097/MD.0000000000001344

**Published:** 2015-10-09

**Authors:** Bei-ge Jiang, Yuan Yang, Hui Liu, Fang-ming Gu, Yun Yang, Lin-Hao Zhao, Sheng-xian Yuan, Ruo-yu Wang, Jin Zhang, Wei-ping Zhou

**Affiliations:** From the Department of Surgery, Eastern Hepatobiliary Surgery Hospital, Second Military Medical University, Shanghai, P.R. China (B-GJ, YY, HL, F-MG, YY, L-HZ, S-XY, R-YW, JZ, W-PZ).

## Abstract

Previous studies showed that suppressor of cytokine signaling 3 (SOCS3) protein is associated with incidence and progression of hepatocellular carcinoma (HCC); however, the association between the genetic polymorphism of SOCS3 gene and HCC remains unknown.

A total of 254 HCC patients and 354 healthy controls were enrolled. All HCC patients underwent partial hepatectomy as initial treatment and were followed. Three SOCS3 gene polymorphisms, namely, rs4969170 A>G, rs8064821 C>T, and rs12953258 C>A were determined.

Our data show that the rs4969170 A>G polymorphism dramatically affects the susceptibility to HCC in our cohorts. Logistic regression analyses revealed that the rs4969170 GG is a risk factor for HCC after the adjustment with confounding factors. The rs4969170A>G polymorphism is also associated with the clinical features of HCC patients and predicts the postoperative relapse-free survival and overall survival. The rs4969170GG genotype carrier had a worse prognosis than the rs4969170AG and rs4969170AA carrier.

Our findings suggest that the rs4969170A>G polymorphism of SOCS3 gene may be used as a prognostic predictor for HCC patients who underwent surgical treatment.

## INTRODUCTION

Hepatocellular carcinoma (HCC) is the fifth most common cancer worldwide and the third cause of cancer-related deaths.^1^ Despite recent advances in cancer treatment, the prognosis of HCC remains very poor. Tumor recurrence rates are more than 70% of cases at 5 years. Some prognostic factors for HCC, such as tumor size, elevated AFP levels, tumor grade, and differentiation status, have been identified.^2^ In addition, some genetic markers predicting HCC prognosis have also been reported.^3^ However, these methods have not proven adequate to predict the prognosis of all HCC patients. Thus, it is important to find a reliable marker for HCC diagnosis and prediction of clinical outcome.

The suppressor of cytokine signaling (SOCS) family proteins has been implicated in the negative regulation of various cytokines.^4^ Recently, emerging evidence suggests that SOCS is a suppressor for several types of cancers, including colon and rectal cancer, lung cancer, prostate cancer, and breast cancer.^5–8^ The SOCS pathway is a key negative regulator of cytokine signaling that inhibits the JAK/STAT signal transduction pathway.^9^ SOCS3 is a major member of SOCS family. The role of SOCS3 in HCC has been reported. SOCS3 is involved in the suppression of tumor growth and metastasis of HCC. Deletion of the SOCS3 gene in liver parenchymal cells promotes hepatitis-induced hepatocarcinogenesis.^10^ SOCS3 silencing is a significant predictor or poor survival, indicating that SOCS3 might play a special role in limiting late-stage HCC progression.^11^

Genetic factors may also play critical roles in the pathogenesis of HCC. Previous studies have demonstrated that genetic variant mainly in the form of single nucleotide polymorphism (SNP) plays an important role in chemosensitivity, tumor recurrence, and prognosis of HCC patients.^12,3^ Although a close association between SOCS3 protein and HCC has been established, the genetic polymorphism of SOCS3 gene and HCC remains unknown.

SOCS3 gene is located in the chromosome region 17q24–17q25 and several gene polymorphisms at different loci have been identified, including rs4969170, rs8064821, and rs12953258. In this study, we enrolled Chinese HCC patients with HC to study whether the above-mentioned SOCS3 polymorphisms can affect the prognosis of HCC patients.

## METHODS

### Patient Enrollment

This is a hospital-based case-control study. A total of 254 patients with primary HCC who underwent a curative liver resection were included in this study between January 2008 and December 2010. The diagnosis was confirmed by pathological examination and/or α-fetoprotein elevation combined with imaging examination (magnetic resonance imaging (MRI) and/or computerized tomography (CT)). The pathological tumor stage was defined according to the sixth edition of the tumor-node-metastasis (TNM) classification of the International Union against Cancer. A total of 354 sex and age-matched healthy volunteers were enrolled as controls. All the participants were unrelated Chinese Han. Institutional ethics committee approval for the project was granted before the study was commenced and was in compliance with the Helsinki Declaration. Written informed consent was obtained from all the patients.

### Treatment and Follow-Up

All patients received the partial hepatectomy as initial therapy and those who previously had chemotherapy or radiation treatment prior to surgery were excluded. The transarterial chemoembolization (TACE) procedure was performed as the postoperative therapy in some patients, if indicated. The regimens used in TACE consisted of carboplatin 300 mg, epirubicin 50 mg, and mitomycin C 8 mg mixed with 5 mL ethiodized oil.^13^ Complete follow-up data were obtained from all HCC patients. Primary study end points were postoperative overall survival (OS) and postoperative relapse-free survival (RFS). OS and RFS were defined as the time from the date of surgery to the date of death from HCC or to the date of local recurrence or detection of distant metastasis, respectively.^14^

### Polymorphisms Selection and Genotyping

We selected 3 SNP variants of SOCS3 gene, namely, rs4969170, rs8064821, and rs12953258 in this study. Genotyping was performed using the 5′-nuclease TaqMan-MGB assay in 384-well plates and the ABI PRISM 7900HT Sequence Detection system (Applied Biosystems, Foster City, California), as previously described by Tang et al.^15^ The raw data were read at the end-point and were clustered into 3 groups that represented the 3 genotypes.

### Western Blotting Analysis

The HCC samples were obtained during the surgical procedure from all 254 HCC patient. Tumor tissue from each patient was lysed with RIPA buffer and protein samples (25 μg) were separated on sodium dodecyl sulfate polyacrylamide gel electrophoresis gels and transferred onto a polyvinylidene difluoride membrane (Bio-Rad Laboratories, Hercules, CA, USA). The membranes were blocked with 5% nonfat dry milk in Tris-buffered saline containing 0.1% Tween 20, and incubated with specific antibodies against SOCS3 (1:200 dilution, Abcam, Cambridge, MA, USA) and β-actin (1:3000 dilution, Millipore, Billerica, MA, USA). Optical densities of the bands were scanned and quantified with the Syngene Gene Tools (Syngene Corp., UK).

### SOCS3 Immunohistological Staining

Formalin-fixed and paraffin-embedded 5-μm-thick tumor sections were deparaffinized, placed in a solution of absolute methanol and 0.3% hydrogen peroxide for 30 min, and treated with blocking serum for 20 min. The slides were incubated overnight with SOCS3 antibody (Abcam, USA) at a 1:200 dilution at 4°. The immune reaction was revealed with 0.06 mmol/L diaminobenzidine (DAB-Dako, DakoCytomation, Carpinteria, California) and 2 mmol/L hydrogen peroxide. Sample scoring was performed by semiquantitative microscopic analysis, measuring the staining area and signal intensity. Both scores were multiplied and the resulting score was used to categorize SOCS3 expression as low (0–6) and high (>6) expressions.^16^

### Statistical Analyses

The Hardy–Weinberg equilibrium was performed to determine the deviation gene allele frequencies by using *χ*^2^ tests in this study. The SOCS3 genotype distributions and allele frequencies were compared by using *χ*^2^ analysis or Fisher's exact test. The multivariate logistic regression analysis was conduced to determine the association of SOCS3 polymorphism and HCC susceptibility, with the adjustment with several conventional risk factors, such as age at enrollment, sex, smoking, and alcohol intake status. For survival comparison, we performed the Kaplan–Meier analyses stratified by the SOCS3 genotypes. The significance of the differences in survival among different genotype carriers was evaluated with the log-rank test. The univariable and multivariable Cox proportional hazards models were conducted to determine the prognostic values of SOCS3 genotype in HCC patients. The hazard ratio (HR) and the 95% confidence interval (95% CI) were calculated. All these above-mentioned statistical analyses were performed by using the SPSS software package (version 16.0; SPSS Inc, Chicago, Illinois). A *P* value less than 0.05 was considered to be statistically significant.

## RESULTS

The characteristics of the study population are presented in Table 1. There was no significant difference in age and gender distribution between HCC and controls. However, HCC group had a significantly higher rate of smoker and heavy alcohol intake compared with controls (*P* < 0.001).

**TABLE 1:**
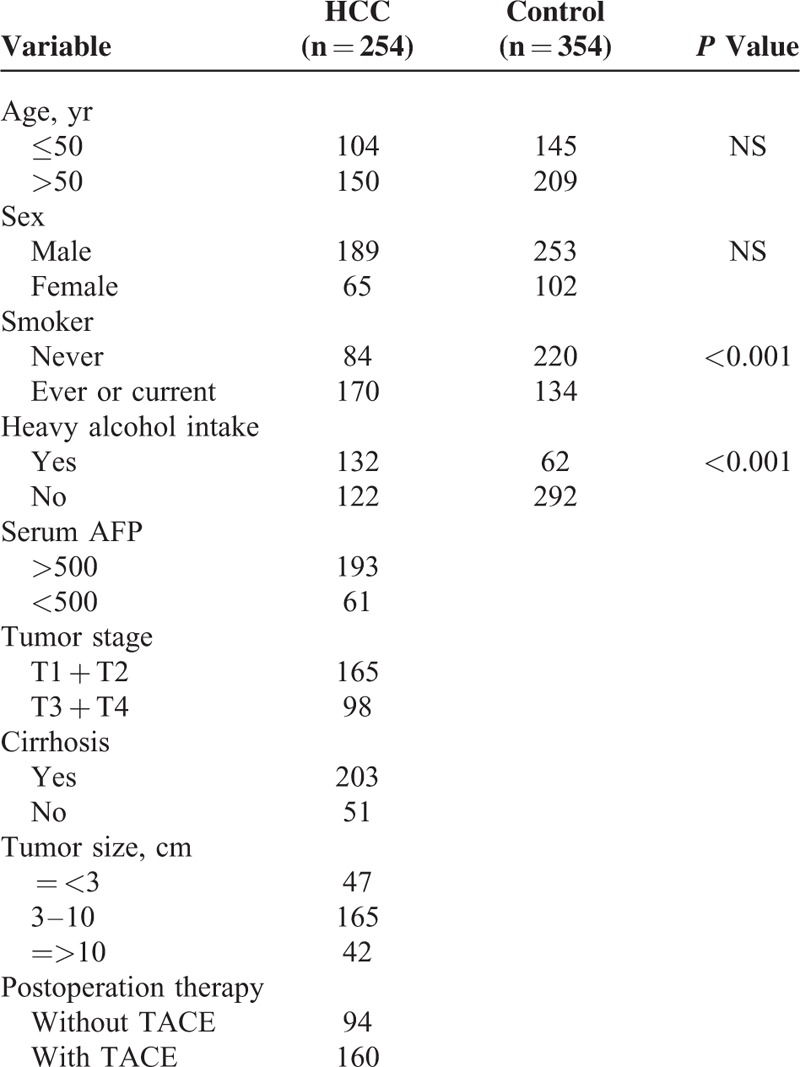
The Characteristics of the Study Population

The genotype frequencies of SOCS3 gene polymorphism in HCC and controls are presented in Table 2. The genotype frequencies of all SNPs in control groups were in Hardy–Weinberg equilibrium (all *P* > 0.05). The genotype frequencies of rs12953258 and rs8064821 were not significantly different between HCC and control groups. For rs4969170 polymorphism, however, the HCC patients had a higher prevalence of GG (38.58%) than control subjects (28.25%, *P* = 0.001). For allele comparison, HCC subjects had lower G allele frequency (62.40%) than controls (54.24%, *P* = 0.001). To determine the risk factor for HCC, we performed the multivariate regression analyses. With rs4969170 AA as reference, the rs4969170 GG carriers had a significantly higher risk for development of HCC after adjustments with age, sex, smoke, and alcohol intake (OR = 1.96, *P* = 0.007). With rs4969170 A allele as reference, the OR for rs4969170 G allele carriage was 1.40 (*P* < 0.001). For SNPs of rs12953258 and rs8064821, the genotype and allele frequencies were not associated with the HCC risk after the adjustment with the aforementioned confounding factors.

**TABLE 2:**
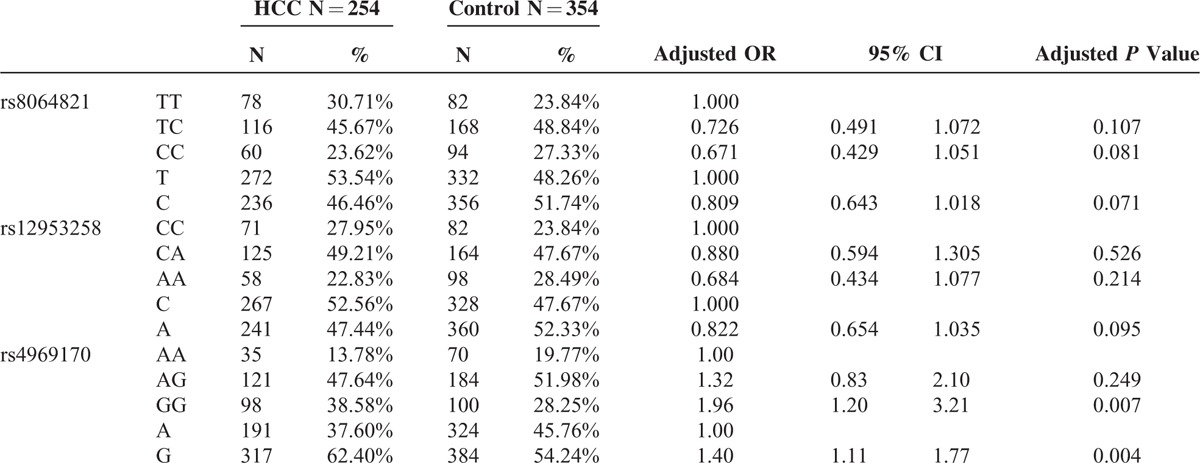
The SOCS3 Genotype Frequencies in HCC Patient and Control Groups

We next analyzed the association between SOCS3 SNPs and the clinicopathological features in all HCC subjects. We found only the rs4969170 was associated with the clinical stage and lymph node metastasis (Table 3). Compared with the rs4969170 AA genotype, the rs4969170 GG genotypes were more frequently appeared in HCC patients with a higher serum AFP level (*P* = 0.004), advanced stage (*P* = 0.023), and cirrhosis rate (*P* = 0.001). The genetic polymorphisms of rs12953258 and rs8064821 did not show association with HCC clinicopathological features (data not shown).

**TABLE 3:**
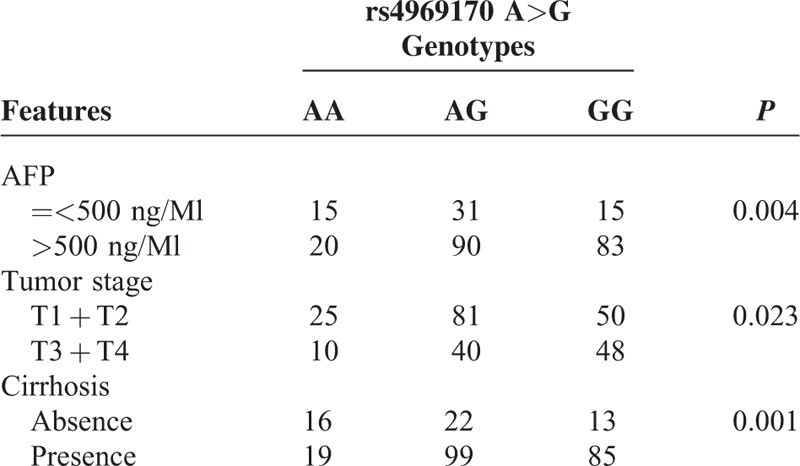
The Association Between the rs4969170 GG Genotypes and the Clinical Features of HCC Patients

**FIGURE 1:**
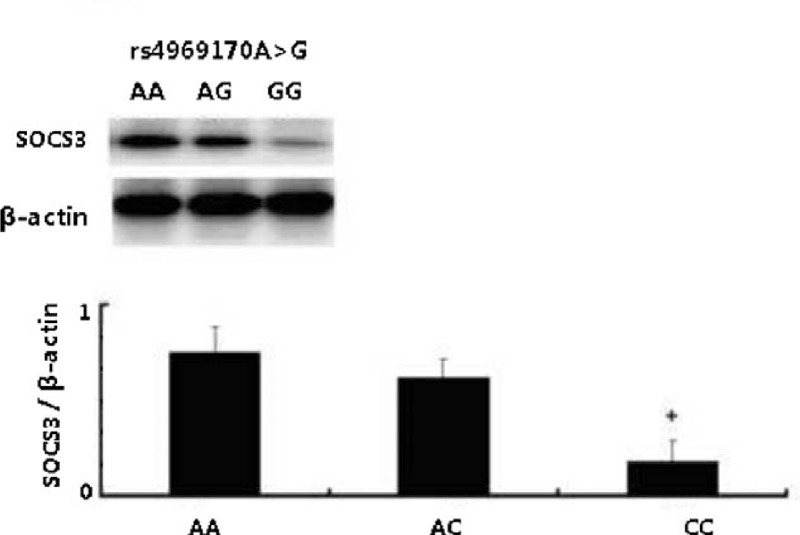
rs4969170A>G and the SOCS3 expression in HCC tumors. The SOCS3 expression n HCC tumor tissues detected by Western blot assay. Patients with rs4969170GG genotypes had a significantly lower SOCS3 expression levels compared with the rs4969170AG and AA carriers.

**FIGURE 2:**
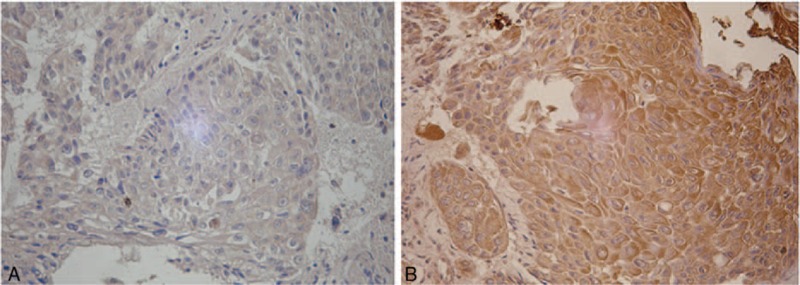
The SOCS3 expression in tumor samples from HCC patients. A, Low SOCS3 expression in an HCC sample. B, High SOCS3 expression in an HCC sample.

All the 254 HCC patients underwent hepatectomy as initial therapies. The tumor tissues were obtained from all HCC patients to detection SOCS3 expressions. The rs4969170AA and the SOCS3 expression in HCC tumors genotype carriers (n = 35) had a significantly reduced SOCS3 expression level compared with the rs4969170AG (n = 121) and GG (n = 98) carriers. The average levels of SOCS3 expression among different genotype carriers are shown in Figure 1. In contrast, the genetic polymorphisms of rs12953258 and rs8064821 were not related to the tumor tissue SOCS3 expression levels (data not shown). The tissue immunohistological staining shows that there are 145 HCC patients who had high SOCS3 expression (immunohistological scores = or >6), while the rest 109 had low SOCS3 expression (immunohistological scores <6). Figure 2 shows the typical images of SOCS3 staining. The SOCS3 expression is not associated with clinicopathological features of HCC patients (data not shown).

**FIGURE 3:**
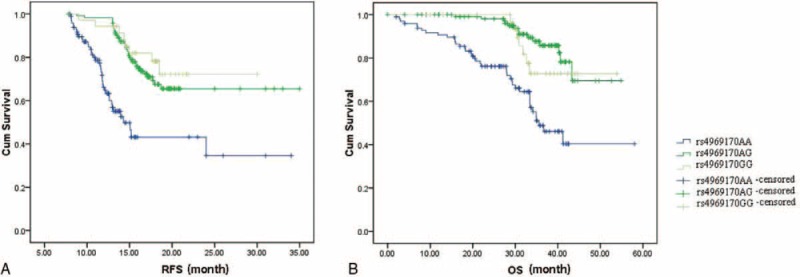
The Kaplan–Miere curves for RFS and OS of HCC patients stratified by rs4969170GG genotypes. A, The mean RFS of patients with the rs4969170AA, AG, and GG genotypes were 18.2 ± 3.6, 17.6 ± 5.2, and 11.7 ± 3.8 months, respectively (log-rank test: *P* = 0.0002). B, The mean OS of patients with the rs4969170AA, AG, and GG genotypes were 36.8 ± 8.2, 35.2 ± 4.8, and 24.5 ± 5.6 months, respectively (log-rank test: *P* < 0.001).

We next analyzed the association between the SOCS3 gene polymorphism and the prognosis of HCC receiving surgical treatment. The 254 HCC patients who underwent hepatectomy were followed for the RFS and OS. A significant correlation between the rs4969170A>G polymorphism and postoperative survival was found. The mean RFS of patients with the rs4969170AA, AG, and GG genotypes were 18.2 ±3.6, 17.6 ± 5.2, and 11.7 ± 3.8 months, respectively (log-rank test: *P* = 0.0002). The mean OS of patients with the rs4969170AA, AG, and GG genotypes were 36.8 ± 8.2, 35.2 ± 4.8, and 24.5 ± 5.6 months, respectively (log-rank test: *P* < 0.001). The Kaplan–Miere curves for RFS and OS of HCC patients stratified by rs4969170GG genotypes are shown in Figure 3A and B. We did not observe the association of tumor SOCS3 expression with the RFS and OS of HCC patients in this study (data not shown).

To determine the predictor factors to the prognosis of HCC patients, the multivariate Cox proportional hazard analysis was then performed, and the variables that showed significance by univariate analysis were adopted as covariates. The clinical characteristics, including age, sex, smoking status, serum AFP, tumor stage, tumor size, and cirrhosis are used as adjustment factor in COX analyses. As shown in Table 4, our data reveal that the rs4969170A>G polymorphism was an independent prognostic factor for RFS (HR = 2.65, 95% CI: 1.98–5.32, *P* < 0.001) and OS (HR = 3.11, 95% CI: 2.43–6.67, *P* < 0.001).

**TABLE 4:**
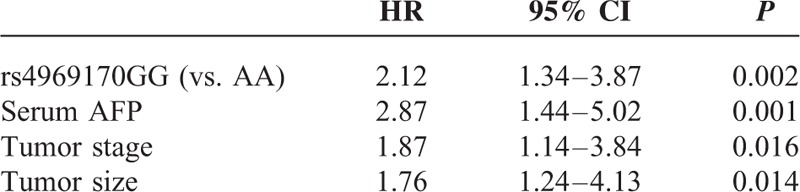
The Prognostic Factors to HCC Patients Receiving Surgical Treatment

## DISCUSSION

In the present study, we investigate the association between 3 common genetic polymorphisms of SOCS3 gene and the clinical outcome of HCC patients receiving surgical treatment as initial therapy. Our data show that the rs4969170A>G polymorphism in the only polymorphism affects the clinical features of HCC patients and predicts the postoperative relapse-free survival and overall survival. The rs4969170GG carriers tend to have a worse prognosis than rs4969170AG and rs4969170AA carriers. These findings suggest that the rs4969170A>G polymorphism of SOCS3 gene may be used as a prognostic predictor for HCC patients who underwent surgical treatment.

HCC is a complex and heterogeneous tumor, the pathogenesis of which probably involves different environmental and genetic factors. The hepatitis B and C viruses are regarded as the major etiological factors associated with the development of HCC, particularly due to their induction of long-term chronic inflammation.^17^ Although surgical resection and liver transplantation could potentially cure HCC, these treatment options are only available to those patients whose tumors are detected early. Therefore, screening and surveillance strategies for HCC have been implemented in clinical guidelines,^18^ but are far from being completely successful due to lack of reliable biomarkers and effective predictive models. To date, several candidate-gene studies have reported associations between SNPs and the presence of HCC.^16,19–22^ Nevertheless, there are limited studies on polymorphisms that affect production of pro-inflammatory molecules and increase the risk of virally induced HCC.

SOCS3 is a member of the suppressor of cytokine signaling family that could inhibit several cytokines such as LIF, IL-6, and IL-11. SOCS3 expression is induced by JAK/STAT signaling and it then binds to specific cytokine receptors.^23,24^ The association between polymorphisms in the SOCS3 gene and various human diseases has been reported. The SNP rs8064821 was associated with approximately 10-month survival advantage compared with noncarriers in patients with resected pancreatic cancer.^25^ In addition, Persico et al claimed a strong association between haplotypes carrying the rs4969170 A allele and resistance to antiviral therapy in patients with chronic hepatitis C.^26^ Although it could be hypothesized that the latter association is related to underlying etiology of HCC, no studies have specifically addressed the role of SOCS3 polymorphisms in HCC so far.

The result presented here showed that subjects with SOCS3 rs4969170 G allele and GG genotype had increased risk of HCC compared with those with genotype AA after adjusting for age, gender, smoking status, and alcohol consumption, and those patients also had larger tumor sizes, higher serum AFP levels, and shorter RFS and OS. We further sought the molecular mechanisms through which rs4969170 GG was associated with HCC. It has been shown that the SNP rs4969170 is located in the promoter region of the SOCS3 gene, which is also the binding region of several transcription factors SNP rs4969170;^26^ therefore, there seems to be a possibility that the allele A/G could influence SOCS3 expression by affecting transcription factor binding.^27^ Indeed, our observations showed the SOCS3 expression was significantly decreased in GG homozygotes. Further investigation of the effect of SNPs on SOCS3 promoter activity will require luciferase assay, which is currently underway. Although there is no evidence indicating that this SNP alters the posttranslational modification of SOCS3, we cannot rule out this possibility. Interestingly, the HCC protective allele A of rs4969170 was associated with higher SOCS expression, which is consistent with previous findings showing reduction of SOCS3 expression was found in HCC patients.^10^

One possible limitation of our study is the relatively small cohort size that may affect the validity of statistical analysis. Additionally, it is necessary to confirm the genetic association in other cohorts, for example, patients and controls who originate from another country. Furthermore, future studies are needed to study other SNPs in or nearby the SOCS3 gene which might be associated with risk for HCC.

In conclusion, we report a striking association between SOCS3 rs4969170 and the clinical characteristics and prognosis of HCC, which is independent of other known risk factors. These data highlight the importance of understanding the roles of SOCS3 genetic polymorphisms to HCC pathogenesis, at least in a Chinese population. If the results of the current study are validated, SOCS3 rs4969170 could potentially be included in a multifactorial risk assessment and also contribute to future preventive or therapeutic strategies targeting HCC.
